# Towards Fault-Tolerant AGV Task Scheduling in Flexible Manufacturing Systems Using a Tree-Based Max-Plus Predictive Approach

**DOI:** 10.3390/s26123898

**Published:** 2026-06-19

**Authors:** Dominik Zaborniak, Paweł Kasza, Marcin Pazera, Marcin Witczak

**Affiliations:** 1 Institute of Control and Computation Engineering, University of Zielona Góra, ul. Prof. Z. Szafrana 2, 65-516 Zielona Góra, Poland; d.zaborniak@stud.uz.zgora.pl (D.Z.); m.pazera@issi.uz.zgora.pl (M.P.); 2 Doctoral School of Exact and Technical Sciences, University of Zielona Góra, ul. Prof. Z. Szafrana 2, 65-516 Zielona Góra, Poland

**Keywords:** fault-tolerant control (FTC), model predictive control (MPC), discrete event system (DES), max-plus algebra, Automated Guided Vehicles (AGVs), Flexible Manufacturing Systems (FMSs), Internet of Things (IoT)

## Abstract

Efficient task assignment for mobile robots is a crucial challenge in modern intralogistics. This paper presents an integrated cyber-physical framework combining predictive tree search on switching max-plus linear systems with a physical IoT-based dispatch interface. The scheduling problem is modelled as a discrete event system, where standard max-plus algebra captures robot synchronization, and a switching mechanism represents alternative resource assignments. To address real-world operational disturbances, the predictive model is enhanced with a fault-tolerant control (FTC) mechanism that dynamically estimates and adapts to non-stationary transport delays. The resulting decision space, which grows exponentially with the prediction horizon, is explored via a predictive tree search algorithm utilizing a quadratic cost function to penalize excessive and uneven transport times. The physical dispatch layer is realized using KIS.BOX IoT devices acting as operator-controlled stations, communicating with the central controller via a WebSocket/STOMP event stream and a lightweight REST API. Simulation results obtained in a Blender 3D environment demonstrate that the proposed FTC predictive strategy significantly reduces the variance of task completion times under fault conditions compared to a baseline First-In-First-Out approach. Furthermore, the IoT integration successfully simulates and validates the feasibility of human-in-the-loop task injection within a realistic, stochastic scenario.

## 1. Introduction

Contemporary industrial and intralogistics environments are undergoing a significant transformation, moving away from static, conveyor-driven setups in favour of highly adaptable and reconfigurable architectures. This shift is largely driven by the increasing market demand for personalized goods, making mass customization a vital strategy to maintain both operational scalability and cost-effectiveness [1]. Such a structural evolution aligns seamlessly with the core principles of Industry 4.0, heavily relying on the deployment of cyber-physical systems, continuous data monitoring, and decentralized, intelligent decision making throughout the entire manufacturing process [2].

The deployment of Automated Guided Vehicle (AGV) fleets plays a foundational role in enabling this high degree of flexibility within internal logistics. Unlike conventional, permanent conveyor belts, autonomous mobile robots provide the capability to dynamically reroute material flows without necessitating any physical alterations to the plant’s layout. According to the extensive survey presented in [3], AGVs now serve as the structural backbone of smart factories. Their implementation deeply affects various operational layers, from basic transport efficiency to advanced production scheduling and overall system architecture. Consequently, these robotic fleets have transitioned from being mere auxiliary transport tools into fundamental elements embedded directly within the central manufacturing control loop.

Parallel to the rapid developments in AGV fleet management, the widespread adoption of Internet of Things (IoT) solutions has opened novel avenues for incorporating human operators into the automated task allocation loop. By utilizing physical IoT endpoints, factory floor workers can effortlessly trigger transport requests, generating real-time data streams that are immediately processed, routed, and translated into actionable scheduling commands [4,5]. Bridging the gap between manual, event-driven IoT inputs and autonomous robotic dispatching constitutes a highly practical challenge that still requires deeper exploration in the current literature.

The task assignment problem for AGV fleets requires deciding which robot transports a given product at a given time. The resulting schedule must minimize transportation time, energy usage, or other process criteria while remaining robust against disturbances and system variability [6,7,8]. Various modelling and control approaches have been proposed in the literature, including mixed-integer linear programming (MILP), Petri nets, automata, and simulation [9,10]. Among these frameworks, max-plus algebra provides a well-established mathematical tool for describing discrete-event systems with synchronization and timing constraints [11]. It has been successfully applied to periodical systems such as railway networks and repetitive production processes, and continues to appear in recent scheduling problems with cyclic behaviour [12].

Classical max-plus models, however, cannot directly represent alternative task assignments or resource choices—capabilities inherent to flexible AGV operation. To address this limitation, switching max-plus linear (SMPL) systems have been introduced, allowing the model to transition between different max-plus representations depending on discrete decisions [13]. Combined with model predictive control (MPC) [14], SMPL systems enable a tree-structured search over possible assignment sequences across a finite prediction horizon. This approach is more flexible than standard MILP formulations, which become cumbersome when nonlinear performance criteria are considered due to the need for piecewise linearization and auxiliary variables [15].

This paper extends the switching max-plus predictive scheduling framework with a physical IoT dispatch layer realized using KIS.BOX devices from the KIS.ME platform. Each KIS.BOX acts as an operator-controlled loading station: the operator selects a target destination by cycling through five colour-coded LED states and confirms the assignment by pressing a button. The resulting task is transmitted in real time to a Python 3.13 script via a WebSocket/STOMP subscription, where it enters a shared task queue and is assigned to one of three mobile robots by the predictive tree search algorithm. Then robot assignments are sent to a plant simulation environment carried out in Blender 3D. The system also incorporates a fault model in which robots are temporarily immobilized for a configurable duration, allowing the scheduling algorithm to be evaluated under realistic disturbance conditions.

The main contributions of this paper are threefold as follows:

1. A switching max-plus fault-tolerant predictive tree search algorithm for AGV task assignment with a nonlinear quadratic cost function.
2. An IoT integration architecture connecting physical KIS.BOX dispatch devices to a 3D simulation via event-driven WebSocket communication and a lightweight REST API.
3. A simulation study in Python/Blender 3D demonstrating reduced variance in task completion times compared to FIFO scheduling, validated under both nominal and fault conditions.

Table 1 explicitly contrasts our proposed method against standard approaches discussed in the paper, highlighting our specific contributions.

The remainder of this paper is organized as follows. Section 2 provides the mathematical background on max-plus algebra and switching systems. It also governs the derivation of the state-space equations for robot synchronization and presents the predictive decision tree construction and cost function. Section 3 describes the system architecture and the IoT integration layer and the Blender 3D simulation environment. Section 4 presents the simulation results and discussion. Section 5 concludes the paper and outlines directions for future work.

## 2. Mathematical Model

### 2.1. Max-Plus Algebra

Max-plus algebra is a highly effective analytical tool for describing discrete event systems (DESs). Its primary advantage lies in its ability to linearize the nonlinear behaviour of concurrent systems, such as waiting for the latest event (synchronization) or parallel task processing. From the perspective of this theory, temporal relations within the system take the form of linear equations, which can be analysed analogously to classical control theory. This algebraic structure is defined over the set of real numbers extended by negative infinity, i.e., $R_{\max}=R\cup \left\{-\infty \right\}$. It operates on two fundamental binary operations: max-plus addition (denoted by the symbol ⊕) and max-plus multiplication (denoted by ⊗). For any two scalars $a,b\in R_{\max}$, these operations are defined as follows:

(1) a⊕b≜max(a,b),

(2) a⊗b≜a+b.

Following standard algebraic convention, max-plus multiplication takes precedence over max-plus addition. The aforementioned operations possess their respective neutral elements. For the addition operation ⊕, the zero element is ε=−∞, since the relation max(a,−∞)=a holds for any value. Conversely, for the multiplication operation ⊗, the identity element is e=0, because a+0=a. Furthermore, scalar exponentiation in this space reduces to classical multiplication, which can be explicitly written as $a^{⊗k}≜k\cdot a$.

These principles scale directly to matrix and vector calculus. Given two matrices $A,B\in R_{\max}^{n\times m}$ and a matrix $C\in R_{\max}^{m\times p}$, matrix addition and multiplication are computed analogously to the rules of classical linear algebra, with the distinction that standard addition is replaced by the max operator, and standard multiplication by standard addition. Consequently, the following definitions hold:

(3) $${\left[A⊕B\right]}_{i,j}=A_{i,j}⊕B_{i,j}=\max\left(A_{i,j},\;B_{i,j}\right),$$

(4) $${\left[A⊗C\right]}_{i,j}=\overset{m}{\underset{k=1}{⨁}}\left(A_{i,k}⊗C_{k,j}\right)=\max_{k=1,\ldots ,m}\left(A_{i,k}+C_{k,j}\right).$$

Similar to standard matrix calculus, matrix multiplication in max-plus algebra is not commutative (i.e., $D_{1}⊗D_{2}\neq D_{2}⊗D_{1}$). This fact is crucial when modelling switching systems, where the sequence of individual dynamic matrices determines the final state of the system. To maintain mathematical precision in iterated multiplication, a convention for writing the cascaded product as a sequence with decreasing indices is introduced:

(5) $$\begin{array}{c} \overset{m}{\underset{i=n}{⨂}}D_{i}≜D_{m}⊗D_{m-1}⊗\cdots ⊗D_{n+1}⊗D_{n},\;\mathrm{for}\;m\geq n. \end{array}$$

The matrix operations are completed by two specific structures: the identity matrix $E_{n}\in R_{\max}^{n\times n}$, whose main diagonal is populated with elements *e* while all off-diagonal positions are ε, and the zero matrix $E_{n}$, filled entirely with ε elements. Exponentiation of a square matrix $D\in R_{\max}^{n\times n}$ is defined as its iterative multiplication by itself:

(6) $$\begin{array}{c} D^{⊗k}=\underset{k\;\mathrm{times}}{\underset{︸}{D⊗D⊗\cdots ⊗D}}. \end{array}$$

Analogous to standard algebra, raising a matrix to the power of zero yields the identity matrix, i.e., $D^{⊗0}=E_{n}$.

### 2.2. System Description

**Figure 1:**
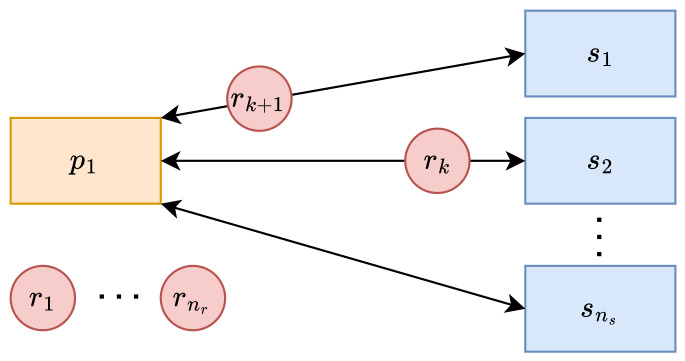
Scheme of the transporting process with a single loading station and multiple destination stations.

The Flexible Manufacturing System (FMS) considered in this study consists of a single loading station, a fleet of mobile transport robots (AGVs), and a set of destination stations. The key assumptions, operation synchronization rules, and their transformation into max-plus algebra are presented below. It is assumed that the system processes a sequence of tasks indexed by a discrete variable k∈N (which also has a meaning of event counter), where u(k) denotes the arrival time of the *k*-th task into the input buffer. A task consists of delivering a product from the loading station to a destination station using one of the mobile robots. The system features a single loading station ($n_{l}=1$), a fleet of $n_{r}$ robots, and a set of $n_{s}$ destination stations, as shown in Figure 1. Each time a robot is dispatched to execute task *k*, it begins by loading the products, which takes time $\tau_{L}$. Then it transports the products to the destination station, which takes $\tau_{t}\left(s,r\right)$ time, unloads the products for $\tau_{U}$ time, and returns to the loading station, taking another $\tau_{t}\left(s,r\right)$ time of driving.

The nominal transport time is determined by the length of the path to the destination station *s*, denoted by d(s), and the speed of the assigned robot *r*, such that $\tau_{t}\left(s,r\right)=d\left(s\right)/v\left(r\right)$. Loading and unloading stations can process at most one robot at a time. Consequently, the model must incorporate rules for their synchronization. The loading operation can only commence when three conditions are simultaneously met:

- The task has entered the system;
- The loading station has finished servicing the previous robot;
- The assigned transport robot has completed its previous task and returned to the loading station.

Let the scalar variable $x_{p}\left(k\right)\in R_{\max}$ denote the time instant at which the loading station becomes available after satisfying the aforementioned conditions. In max-plus algebra, the conditions take the form:

(7) $$\begin{array}{c} x_{p}\left(k\right)=\left(x_{p}\left(k-1\right)⊕x_{r_{k}}\left(k-1\right)⊕u\left(k\right)\right)⊗\tau_{L}, \end{array}$$

where $x_{r_{k}}\left(k-1\right)$ denotes the availability time instance of robot $r_{k}$ as given by the system state after step k−1. It should be noted that this robot did not necessarily participate in the execution of task k−1; in such cases, this variable carries forward its historical availability time from the completion of its last actual activity. For task *k*, the system’s evolution is fully determined by the choice of destination station $s_{k}≜s\left(k\right)\in \left\{1,\ldots ,n_{s}\right\}$ and the robot assignment $r_{k}≜r\left(k\right)\in \left\{1,\ldots ,n_{r}\right\}$. The choice of destination station is dictated by the specific order *k* and the operator requesting it. Conversely, the selection of the robot sequence $\left\{r_{k},\;r_{k+1},\;r_{k+2},\ldots ,r_{k+N_{u}}\right\}$ constitutes the decision variables for allocating these shared resources within the system. The overall performance of the system relies heavily on this specific selection. Let the state vectors $x_{s}\left(k\right)\in R_{\max}^{n_{s}\times 1}$ and $x_{r}\left(k\right)\in R_{\max}^{n_{r}\times 1}$ represent, respectively, the availability time instances of all destination stations and all robots immediately after servicing task *k*. The evolution of $x_{s}\left(k\right)$ involves a selective update of only the component with index $s_{k}$ for event-step *k*. The remaining vector elements retain their values from the previous step, reflecting the absence of changes in their state. The new value $x_{s_{k}}\left(k\right)$ results from the synchronization between the previous occupancy state of this station and the moment the new goods are delivered and unloaded by robot $r_{k}$. This moment depends directly on the loading completion time $x_{p}\left(k\right)$, the transport time, and the unloading time $\tau_{U}$:

(8) $$\begin{array}{c} x_{s}\left(k\right)=\left[\begin{array}{c} x_{s_{1}}\left(k-1\right)\\ \vdots \\ x_{s_{k}}\left(k-1\right)⊕\left(x_{p}\left(k\right)⊗\tau_{t}\left(s_{k},r_{k}\right)⊗\tau_{U}\right)\\ \vdots \\ x_{s_{n_{s}}}\left(k-1\right) \end{array}\right] \end{array}$$

Similarly, the update of the fleet availability vector $x_{r}\left(k\right)$ occurs only for robot $r_{k}$, which, after completing the unloading at station $s_{k}$, must make the return journey to become available again at the loading station:

(9) $$\begin{array}{c} x_{r}\left(k\right)=\left[\begin{array}{c} x_{r_{1}}\left(k-1\right)\\ \vdots \\ x_{r_{k}}\left(k-1\right)⊕\left(x_{s_{k}}\left(k\right)⊗\tau_{t}\left(s_{k},r_{k}\right)\right)\;\\ \vdots \\ x_{r_{n_{r}}}\left(k-1\right) \end{array}\right] \end{array}$$

Since each assignment decision $\left(s_{k},r_{k}\right)$ defines a unique evolution path for the entire system state, it demonstrates that the considered AGV fleet management system belongs to the class of switching max-plus-linear systems (SMPLSs). By defining the full state vector $x\left(k\right)={\left[x_{p}\left(k\right)\;x_{s}{\left(k\right)}^{T}\;x_{r}{\left(k\right)}^{T}\right]}^{T}\in R_{\max}^{(1+n_{s}+n_{r})\times 1}$ and combining the aforementioned synchronization rules (7)–(9) into a state equation familiar from classical control theory, the system’s evolution can be expressed as:

(10) x(k)=A(k)⊗x(k−1)⊕B(k)⊗u(k).

The structure of the state matrix $A\left(k\right)\in R_{\max}^{\left(1+n_{s}+n_{r}\right)\times \left(1+n_{s}+n_{r}\right)}$ and the input matrix $B\left(k\right)\in R_{\max}^{(1+n_{s}+n_{r})\times 1}$ depends directly on the decision variables $\left(s_{k},r_{k}\right)$ chosen at step *k*. Let us define the auxiliary variables:

- $\alpha =\tau_{L}⊗\tau_{t}\left(s_{k},r_{k}\right)⊗\tau_{U}$,
- $\beta =\alpha ⊗\tau_{t}\left(s_{k},r_{k}\right)$,

Then the system matrices take the following block form:

(11) $$A\left(k\right)=\left[\begin{array}{ccc} \tau_{L} & E & A_{pr}\left(k\right)\\ A_{sp}\left(k\right) & E_{\tau_{U}}\left(k\right) & A_{sr}\left(k\right)\\ A_{rp}\left(k\right) & A_{rs}\left(k\right) & E_{\beta }\left(k\right) \end{array}\right],\;B\left(k\right)=\left[\begin{array}{c} \tau_{L}\\ A_{sp}\left(k\right)\\ A_{rp}\left(k\right) \end{array}\right]$$

where E denotes a zero matrix (of dimensions $1\times n_{s}$). The remaining matrices in the model (10) act as sparse switching operators, whose elements are ε except for specific positions resulting from the assignments $s_{k}$ and $r_{k}$:

- Vectors: ${\left[A_{pr}\left(k\right)\right]}_{1,r_{k}}=\tau_{L},\;{\left[A_{sp}\left(k\right)\right]}_{s_{k},1}=\alpha ,\;{\left[A_{rp}\left(k\right)\right]}_{r_{k},1}=\beta$;
- Sparse matrices: ${\left[A_{sr}\left(k\right)\right]}_{s_{k},r_{k}}=\alpha ,\;{\left[A_{rs}\left(k\right)\right]}_{r_{k},s_{k}}=\tau_{U}⊗\tau_{t}\left(s_{k},r_{k}\right)$;
- Modified identity matrices: $E_{\tau_{U}}\left(k\right)$ and $E_{\beta }\left(k\right)$ are standard max-plus identity matrices *E* with their $\left(s_{k},s_{k}\right)$ and $\left(r_{k},r_{k}\right)$ diagonal elements replaced by $\tau_{U}$ and β, respectively.

The output of the system is the completion time of unloading product *k* at a given destination station $s_{k}$. This is represented by the equation:

(12) $$\begin{array}{cc} y(k) & =C(k)⊗x(k) \end{array}$$

The output matrix takes the form:

(13) $$C\left(k\right)=\left[\begin{array}{ccc} \varepsilon & C_{s}\left(k\right) & E \end{array}\right],$$

where $C_{s}\left(k\right)\in R_{\max}^{1\times n_{s}}$ contains the max-plus identity element *e* at position $s_{k}$ and ε elsewhere, while E is a $1\times n_{r}$ zero matrix.

### 2.3. Model Predictive Control

Model predictive control (MPC) constitutes an effective framework for managing complex dynamic systems. Its primary strength lies in the ability to simulate the future behaviour of the plant based on its mathematical model, enabling the system to anticipate upcoming events and dynamically adapt to changing operational conditions. In the context of AGV fleet management, the MPC algorithm determines the optimal sequence of resource assignments with respect to a cost function *J* at each decision step, analysing the potential impact of control actions over a predefined prediction horizon of length $N_{p}$. Using the state Equation (10) derived in the previous section, the evolution of the system can be evaluated analytically. Assuming knowledge of the current state vector x(k) and the known (or estimated) arrival times of future tasks u, the predicted system state at a future step k+p (where $p\in \{1,\ldots ,N_{p}\}$) is expressed by the following equation in max-plus algebra:

(14) $$\begin{array}{c} \begin{array}{cc} x(k+p) & =\left(\overset{p-1}{\underset{i=0}{⨂}}A\left(k+i\right)\right)⊗x\left(k\right)\overset{p}{\underset{j=1}{⨁}}\left(\overset{p-1}{\underset{i=j}{⨂}}A\left(k+i\right)\right)⊗B\left(k+j-1\right)⊗u\left(k+j\right)\\ y(k+p) & =C(k)\;x(k+p) \end{array} \end{array}$$

The primary objective of the optimization is to select a resource assignment sequence

(15) $$\begin{array}{c} \pi_{p}=\left\{r_{k+p-1},\;r_{k+p-2},\;\ldots \;,r_{k+1},\;r_{k}\right\} \end{array}$$

that defines the switching sequence for matrices A(k) and B(k) in such a way that minimizes the specified cost function over the entire prediction horizon $N_{p}$, i.e., $\min J(k+N_{p})$. In the context of intralogistics systems, this function typically penalizes excessive total system delays or the variance of delivery times. According to the receding horizon principle, once the optimal sequence $\pi_{p}$ is determined, only the first control decision is implemented. Upon processing of the next task, the discrete step increments (k→k+1), and the optimization process is repeated. Because the analysed FMS belongs to the class of switching systems, the decision space naturally expands into a tree structure over the prediction horizon. Each node in this search tree corresponds to an alternative assignment decision $\left(s_{k},r_{k}\right)$, generating branches at subsequent prediction levels. A direct exploration of this structure allows for the evaluation of costs at its leaf nodes.

Solving the optimization problem within this tree structure relies on an exhaustive search method. The major advantage of this approach is the ability to employ any nonlinear cost function. Mixed-Integer Linear Programming (MILP) solvers are widely used in the context of max-plus algebra, but they strictly require linear performance indices. In contrast, the tree-based search approach permits the direct application of arbitrary, nonlinear objective functions. However, the main limitation of this method is the combinatorial explosion of possible states. The size of the decision space grows exponentially with the prediction horizon, leading to a computational complexity of $O(n_{r}^{N_{p}})$. Consequently, for long prediction horizons, the application of a direct search algorithm becomes computationally prohibitive. Nevertheless, in the considered manufacturing system, the implementation of an exhaustive search is fully justified and practical due to the specific nature of the logistics process. Transport tasks often appear stochastically, triggered by an operator manually calling a robot from a station. Under these conditions, the system lacks long-term deterministic knowledge of future orders. Therefore, it is reasonable to assume that the maximum look-ahead for incoming requests is limited to a relatively short horizon (e.g., $N_{p}\leq 8$ steps). With such a restricted prediction horizon, the exponential growth of the tree does not pose a critical technological barrier. The exhaustive search method remains highly efficient computationally, enabling real-time decision optimization without the risk of converging to local minima.

### 2.4. Fault-Tolerant Control

The effectiveness of the MPC strategy depends on the accuracy of the mathematical model. In real-world FMS, nominal parameters rarely remain constant. Factors such as the mechanical wear of components, robot battery voltage drops, or dynamic obstacles on transport routes introduce delays in travel times. Any discrepancy between the model prediction and the actual state degrades scheduling quality. Over a prediction horizon, this can lead to the identification of false minima in the objective function. To make the algorithm robust against these phenomena, the predictive model has been extended with a fault-tolerant control (FTC) mechanism. Following the methodology presented in the study [16], a fault *f* in a max-plus system is defined as an additive time delay, representing a deviation from the system’s nominal parameters. This definition of a fault directly affects the structure of the dynamic matrices by altering the duration of specific operations. To incorporate uncertainty into the decision-making process, a matrix of estimated delays $F\in R_{\max}^{n_{r}\times n_{s}}$ is introduced. This matrix aggregates the current knowledge regarding transport disturbances for each station–robot pair:

(16) $$\begin{array}{c} F=\left[\begin{array}{cccc} \tau_{f}\left(1,1\right) & \tau_{f}\left(2,1\right) & \ldots & \tau_{f}\left(n_{s},1\right)\\ \tau_{f}\left(1,2\right) & \ddots & \; & \vdots \\ \vdots & \; & \ddots & \vdots \\ \tau_{f}\left(1,n_{r}\right) & \ldots & \ldots & \tau_{f}\left(n_{s},n_{r}\right) \end{array}\right] \end{array}$$

where the element $\tau_{f}\left(s,r\right)$ represents the additional travel time estimated for robot *r* servicing a task directed to station *s*. Consequently, the actual travel time utilized by the search algorithm, denoted as ${\hat{\tau }}_{t}$, becomes the sum of the nominal value and the fault-induced correction, expressed via max-plus multiplication:

(17) $${\hat{\tau }}_{t}\left(s,r\right)=\tau_{t}\left(s,r\right)⊗\tau_{f}\left(s,r\right).$$

The delay estimate $\tau_{f}\left(s,r\right)$ is updated recursively upon the completion of each transport task, allowing the system to continuously adapt to the current characteristics of the environment. This is achieved using an exponential forgetting update rule (evaluated in conventional algebra):

(18) $$\tau_{f}^{k+1}\left(s,r\right)=\alpha \;\tau_{f}^{k}\left(s,r\right)+\left(1-\alpha \right)\;e^{k}\left(s,r\right),$$

where $e^{k}\left(s,r\right)$ denotes the innovation signal. This signal is defined as the difference between the measured return time of the dispatched robot for task *k*, denoted by ${\tilde{x}}_{r_{k}}\left(k\right)$, and the value predicted by the model: $e^{k}\left(s,r\right)={\tilde{x}}_{r_{k}}\left(k\right)-x_{r_{k}}\left(k\right)$. The tuning parameter α∈[0,1] determines the adaptation speed of the model. Lower values of α enable a faster response to sudden congestions, while higher values provide greater robustness against momentary measurement noise.

During the evaluation of the search tree over the specified horizon $N_{p}$, the current delay estimate remains constant. Therefore, the system dynamics matrices in Equation (14) take a form that depends on the current knowledge of faults, i.e., A(k+i,F(k)) for i∈{0,…,p−1}.

### 2.5. Problem Statement

In summary, the optimization problem can be described by:

- Inputs (Parameters):
  − Task arrival times u(k).
  − Nominal transport times $\tau_{t}\left(s,r\right)$.
  − Loading $\tau_{L}$ and unloading $\tau_{U}$ durations.
  − Current availability state of loading station $x_{p}\left(k\right)$, destination stations $x_{s}\left(k\right)$, and robots $x_{r}\left(k\right)$.
  − Current estimated fault delays *F*.
- Outputs (Decision Variables): The optimal sequence of robot resource assignments $\left\{r_{k},r_{k+1},\ldots ,r_{k+N_{p}}\right\}$ over the prediction horizon. (Note: The destination station $s_{k}$ is dictated by the operator’s input, not the algorithm).
- Objective: To determine an assignment sequence that minimizes a nonlinear quadratic cost function $J_{2}=\sum {\left(y\left(k+i\right)-u\left(k+i\right)\right)}^{2}$ over the prediction horizon $N_{p}$. This specific objective heavily penalizes large individual delays to prevent task “starvation” and ensures a balanced fleet workload.

### 2.6. Comparison of Computational Burden: MILP vs. Proposed Tree-Based Approach

The computational characteristics of Mixed-Integer Linear Programming (MILP) versus the proposed tree-based predictive approach can be compared as follows:

Mixed-Integer Linear Programming (MILP)

- Linearity constraints: MILP solvers are widely used in the context of max-plus algebra but strictly require linear performance indices (such as the classic linear criterion $J_{1}=\Sigma \left(y-u\right)$).
- Burden with nonlinear criteria: When nonlinear performance criteria are considered (such as the quadratic cost function $J_{2}$ needed to penalize excessive individual delays), standard MILP formulations become cumbersome. Handling these nonlinearities requires piecewise linearization and the introduction of auxiliary variables, which significantly complicates the model.

Proposed Approach (Tree-Based Predictive Search)

- Flexibility with nonlinear criteria: The major advantage of the tree-based exhaustive search approach is its ability to permit the direct application of arbitrary, nonlinear objective functions without requiring linearization workarounds.
- Combinatorial explosion: The main limitation of this method is the combinatorial explosion of possible states. The size of the decision space grows exponentially with the prediction horizon, leading to a computational complexity of $O(n_{r}^{N_{p}})$, where $n_{r}$ is the fleet size and $N_{p}$ is the prediction horizon.
- Feasibility limits: For long prediction horizons, the application of a direct search algorithm becomes computationally prohibitive.
- Practical efficiency: In the considered stochastic intralogistics systems, the system lacks long-term deterministic knowledge of future orders, making a restricted prediction horizon (e.g., $N_{p}\leq 8$ steps) entirely reasonable. With such a restricted horizon, the exponential growth of the tree does not pose a critical technological barrier. The exhaustive search method remains highly efficient computationally, enabling real-time decision optimization without the risk of converging to local minima.

## 3. System Architecture

To verify the effectiveness of the proposed fault-tolerant control algorithm, a hybrid simulation environment was developed. This environment combines physical industrial interfaces with a virtual logistics model. It was designed to fully reflect the mathematical structure of the model predictive control (MPC) framework:

- Physical KIS.BOX devices (Input generators): These act as a stochastic event generator within the system. A button press by an operator determines the physical arrival time of a new task. This action directly dictates the construction of the input vector u(k) and the assignment of the destination station $s_{k}$ to the task. The absence of a predefined schedule tests the algorithm under conditions of complete uncertainty (on-demand operation).
- Blender 3D virtual environment (Controlled plant): This serves as the Digital Twin of the production hall, acting as the physical plant. It is where the decision sequence $\pi_{p}$ determined by the optimizer is executed. Crucially, this environment is responsible for measuring and returning the actual task completion times achieved by the robots, denoted as ${\tilde{x}}_{r_{k}}\left(k\right)$. This feedback enables the calculation of the prediction error e(k) and the subsequent update of the fault matrix *F* in accordance with the FTC mechanism.
- Python-based decision module (Central controller): This acts as the main processing unit, continuously gathering data from the KIS.ME hardware layer (the input vector u) and the Blender 3D environment (the measurements $\tilde{x}$). At each discrete step *k*, it evaluates the search tree to determine the optimal resource allocation.

### 3.1. Introduction to the KIS.ME Platform

In the era of the Industry 4.0 paradigm, modern enterprises operating on a global scale are subject to constant cost pressure and the imperative to increase operational efficiency [2,17]. This phenomenon drives the need for continuous process optimisation, as well as ensuring their measurability and transparency, both in the areas of production and logistics. In response to these challenges, digital solutions based on the concept of the Internet of Things (IoT) are gaining increasing importance, enabling the automated acquisition and real-time analysis of data [4,5,18].

The Internet of Things is defined as a system of interconnected devices and objects equipped with unique identifiers, capable of communicating and exchanging data without direct human intervention [4]. The integration of IoT technologies with industrial systems leads to the creation of cyber-physical environments, in which operational data is processed continuously and used to support optimisation decisions [19].

The KIS.ME (Keep It Simple. Manage Everything) platform fits within this paradigm, constituting a comprehensive IoT-class solution designed to simplify the digitalisation process in the areas of production and logistics. The system integrates the activities of people and machines, enabling effective data-driven process management.

The platform architecture encompasses both a hardware layer and a software layer built on cloud infrastructure. The hardware components facilitate the acquisition of process data, while the software layer is responsible for its processing, analysis, and visualisation.

The central element of the system is the KIS.MANAGER environment, which serves as the primary communication node responsible for orchestrating data flow and implementing business logic.

A key capability of the platform is the ability to create Digital Twins—virtual representations of physical assets that enable their real-time monitoring and analysis [17,20,21]. The application of this concept supports predictive maintenance and the optimisation of production processes.

The platform also enables the definition of Key Performance Indicators (KPIs) and the automated calculation of Overall Equipment Effectiveness (OEE), one of the fundamental performance metrics in manufacturing environments [22,23]. The integration of these mechanisms with IoT systems allows for the ongoing identification of bottlenecks and the dynamic improvement of processes [24].

Communication security and reliability are ensured through the use of the lightweight MQTT protocol, widely adopted in IoT systems owing to its efficiency and low bandwidth requirements [25,26].

**Figure 2:**
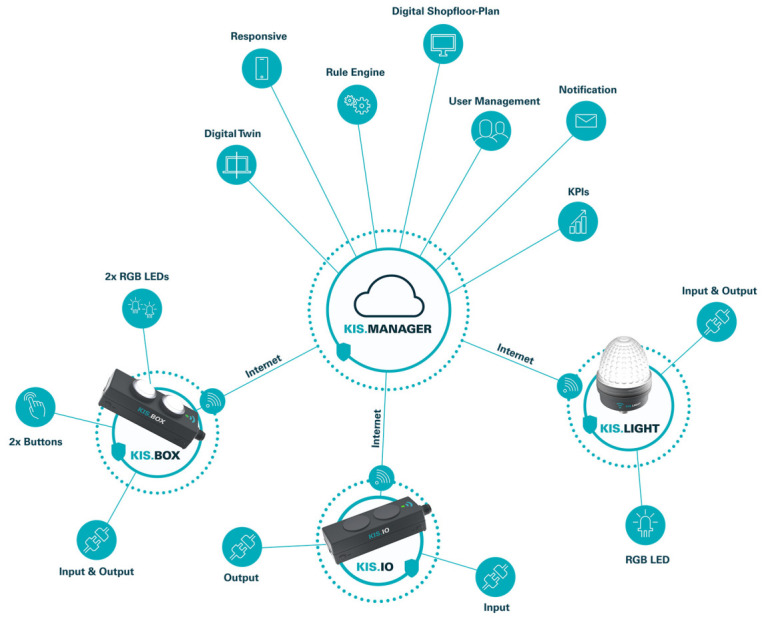
The KIS.ME platform [17].

As a result, the KIS.ME platform supports the transformation of enterprises towards Smart Factories, in which production processes are subject to continuous monitoring, analysis, and data-driven optimisation [17,19]. The architecture of the KIS.ME platform is presented in Figure 2.

In the presented system, each KIS.BOX device represents a dedicated dispatch point within the warehouse environment. An operator selects a target loading station by cycling through five predefined LED colours using button 1, then confirms the task assignment by pressing button 2. The confirmed task is immediately forwarded to the central task queue in KIS.MANAGER and subsequently assigned to an available transport robot in the Blender 3D simulation.

**Figure 3:**
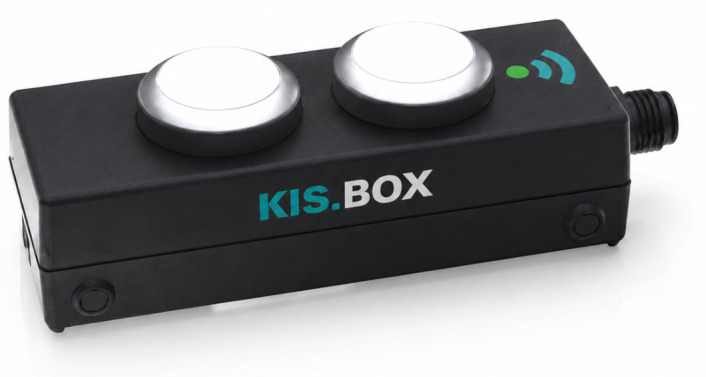
The KIS.BOX device [17].

The KIS.BOX presented in Figure 3 is a universal Human–Machine Interface (HMI) designed in the form of a compact, industrially rated enclosure equipped with two function buttons. Its wireless connectivity and dual power supply options make it suitable for flexible deployment across warehouse and production environments without dedicated cabling infrastructure. Table 2 summarizes the main technical specifications of the KIS.BOX device.

Each KIS.BOX device is equipped with a Status LED and an Operational LED. The Status LED reflects the device connection state, while the Operational LED is fully configurable and serves as the primary feedback channel for the operator.

In the presented scenario, five of the eight available LED colours are actively used, each mapped to a specific loading station in the warehouse simulation. Button 1 cycles through these five colours sequentially, allowing the operator to visually select the desired destination before confirming the task. The remaining three colour slots are reserved for future extension of the station layout. All colour values are represented numerically in the KIS.ME API, as detailed in Table 3.

The two button roles are fixed throughout the scenario and do not change between operators or sessions:

- Button 1—cycles the Operational LED to the next active colour in the sequence, allowing the operator to select the target loading station;
- Button 2—confirms the selection, creates a task entry in the queue, switches the LED to flashing mode as an acknowledgement signal, and locks the interface until the assigned robot completes the transport cycle.

The Rule Engine in KIS.MANAGER enables the definition of IF–THEN dependencies, transforming physical events into system responses.

- Triggers—initiate the evaluation of a rule;
- Conditions—logical premises (AND/OR);
- Actions—operations executed once the conditions are met.

### 3.2. Task Selection and Execution Logic

The control process can be expressed as Algorithm 1.
**Algorithm 1** User interaction handling in KIS.BOX  1: Set initial LED state = WHITE  2: queue←∅  3: **while** system is active **do**  4:     **if** button 1 is pressed **then**  5:          Switch LED to the next colour in the sequence  6:     **end if**  7:     **if** button 2 is pressed **then**  8:          task← current LED colour  9:          Add task to queue10:          Set LED to flashing green11:          Lock button12:     **end if**13:     **if** task completed **then**14:          Unlock button15:          Set LED = WHITE16:     **end if**17: **end while**

Target selection is carried out by cyclically toggling the LED states. Each colour corresponds to a specific location within the simulation environment.

Confirming a task results in:

- The task being added to the queue;
- The LED switching to flashing mode;
- The input interface being locked.

Upon completion of a task:

- The system receives a feedback signal;
- The device state is reset;
- A new cycle can be initiated.

### 3.3. Communication Between Blender 3D and KIS.ME

The integration between the Blender 3D simulation environment and the KIS.ME platform is implemented via two complementary communication mechanisms: a REST API for synchronous request–response interactions, and a WebSocket connection using the STOMP protocol for asynchronous, real-time event streaming. This dual-channel architecture ensures both reliable command dispatch and low-latency state synchronisation between the physical KIS.BOX devices and their virtual counterparts in the simulation.

### 3.4. Communication Architecture

The fundamental unit of data exchange within the KIS.ME platform is the datapoint—a named, typed value associated with a specific asset, reflecting either its current state or accepting commands from the platform. The integration relies on two complementary mechanisms: a REST API for synchronous request–response interactions, and a WebSocket connection using the STOMP protocol for asynchronous, real-time event streaming.

The REST API follows standard HTTP conventions with Bearer token authentication. Two primary endpoints are used: GET/assets/{id}/datapointsretrieves current datapoint values during initialisation, while POST/assets/{id}/datapoints issues commands from the simulation to the device. The datapoints relevant to this integration, together with their directions and types, are listed in Table 4.

Real-time updates from KIS.BOX devices are delivered to the Blender client via a WebSocket connection using the STOMP (Simple Text Oriented Messaging Protocol) framing layer. This mechanism allows the simulation to react immediately to physical button presses without polling.

**Figure 4:**
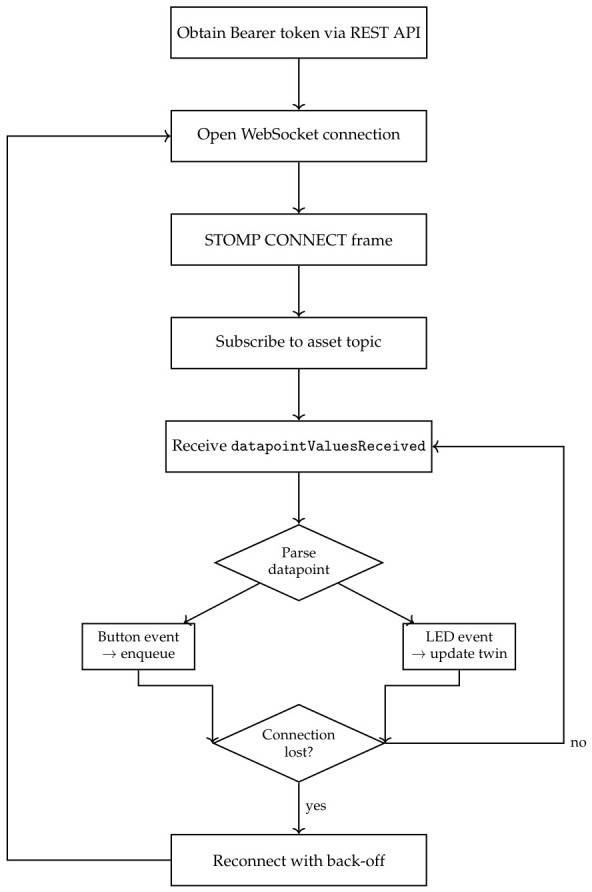
Full WebSocket/STOMP communication sequence with error recovery. Arrows indicate the direction of process flow, while “yes” and “no” denote decision outcomes.

The full communication sequence is presented in Figure 4.

An example STOMP message payload delivered upon a button press event is structured as follows:

{

“assetId”: “abc123”,

“datapointId”: “button2”,

“value”: true,

“timestamp”: “2026-04-28T12:00:05Z”

}

### 3.5. Event-to-Action Mapping

Upon receiving a datapointValuesReceived event, the Blender integration layer evaluates the datapointId field and dispatches the appropriate response. The mapping between incoming events and simulation actions is defined in Table 5.

#### Error Handling and Reconnection Strategy

Network interruptions are handled by the Blender client through an exponential back-off reconnection strategy. If the WebSocket connection is lost, the client waits an initial interval of 1 second before attempting to reconnect, doubling the interval on each successive failure up to a maximum of 30 s. Once reconnected, the client re-authenticates, re-subscribes to all asset topics, and issues a GET request to resynchronise the current datapoint state, ensuring no event-driven state inconsistencies accumulate during the outage.

### 3.6. Simulation Logic and Task Management

**Figure 5:**
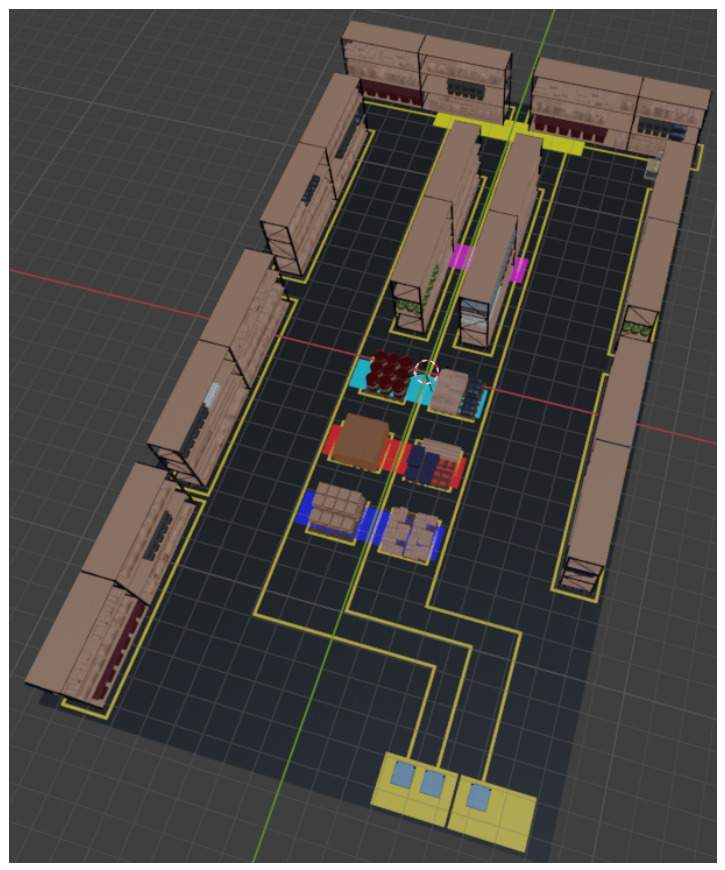
Simulated warehouse environment of the intralogistics system rendered in Blender 3D.

The simulation model represents a simplified intralogistics system in which mobile robots carry out transport tasks between defined points within the simulation workspace, as illustrated in Figure 5.

### 3.7. Simulation Model and Robot Behaviour

The simulation represents a simplified intralogistics environment comprising three autonomous transport robots, a shared task queue, and five target stations mapped to distinct LED colours (cf. Table 3). Each station corresponds to a fixed coordinate in the simulation space; the white colour designates the loading station, while red, blue, green, yellow, and purple identify unloading stations 1 through 5 respectively.

**Figure 6:**
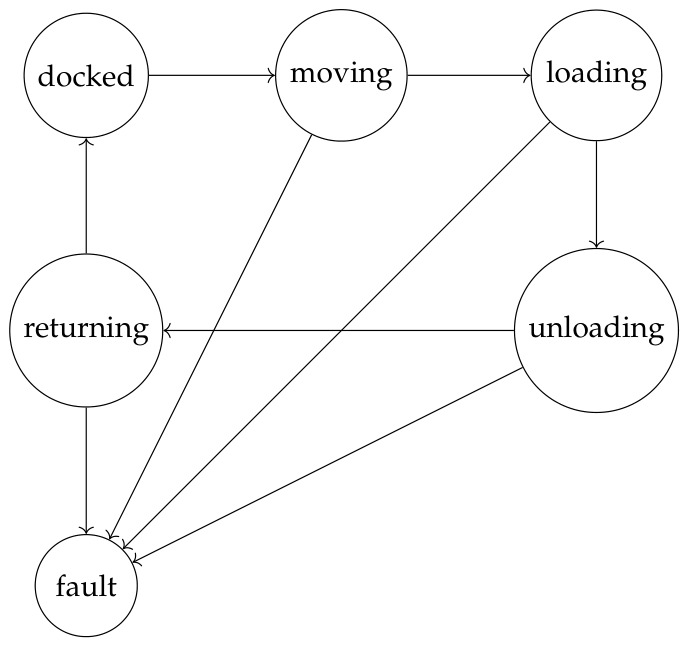
Robot state diagram. Arrows indicate transitions between states in the finite state machine.

Robot behaviour is modelled as a Finite State Machine (FSM), whose states and transitions are illustrated in Figure 6. The meaning of each state is summarised in Table 6.

### 3.8. Task Management

**Figure 7:**
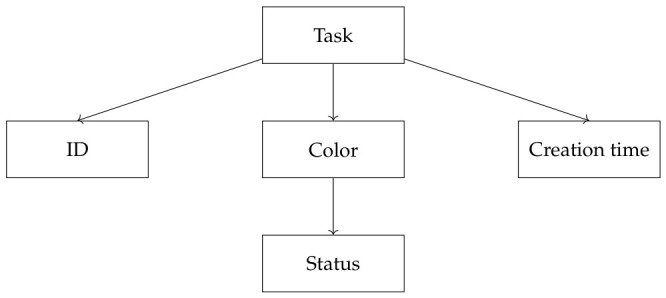
Task structure within the system.

Each task is represented as a data record comprising a unique identifier, the target station colour, a creation timestamp, and a current execution status (cf. Figure 7). Tasks are generated by operators via the KIS.BOX interface and inserted into a shared queue, which supports concurrent access from multiple operators simultaneously.

**Figure 8:**
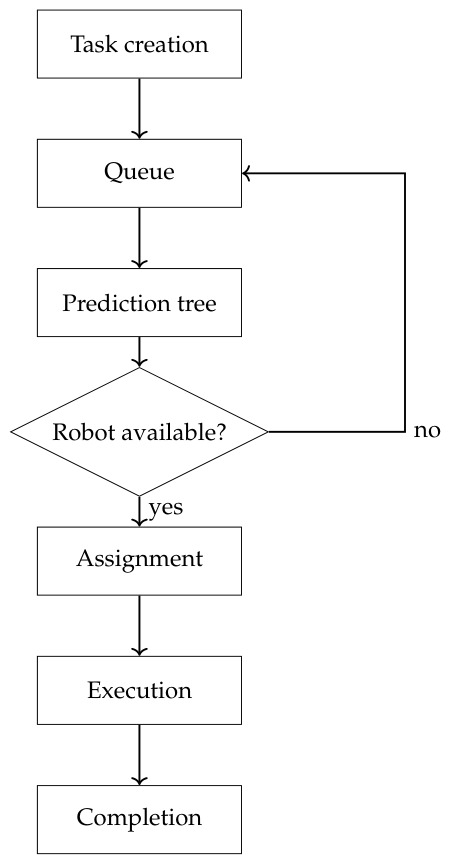
Task lifecycle accounting for robot availability. Arrows indicate the flow of the process between lifecycle stages.

Assignment of a queued task to a robot is governed by three criteria evaluated jointly: the robot must be in the docked state, it must not be in a fault condition, and among all eligible robots the one minimising travel distance and current workload is selected. If no robot satisfies these criteria at the time of task creation, the task remains in the queue until a robot becomes available, as depicted in the lifecycle diagram (Figure 8).

The shared queue architecture inherently supports multi-operator scenarios: tasks generated simultaneously by different operators are serialised into a single ordered structure, with optional priority weighting available for time-critical assignments.

The task assignment criteria are formally defined in Table 7.

The multi-operator characteristics of the shared queue are summarised in Table 8.

### 3.9. System Architecture Overview

The presented system integrates three distinct layers: a 3D visualisation of mobile robots (AGVs) rendered in Blender 3D, physical KIS.BOX devices representing loading stations, and a lightweight HTTP API enabling control and monitoring of the entire system from an external decision tree. A defining characteristic of this architecture is data minimalism—KIS.BOX devices transmit only button press events, while the Blender 3D API exposes a small set of discrete robot states rather than full motion trajectories.

All robot logic runs as a single script executed directly inside Blender 3D. The script performs the following initialisation steps at startup:

- Defines three robots (01.robot–03.robot), each with its own path represented as an ordered list of Vector waypoints in 3D space;
- Launches an HTTP server on port 8080 in a dedicated thread (threading.Thread);
- Launches a WebSocket client connecting to the KIS.ME API in a second dedicated thread.

The system does not poll KIS.BOX devices for their current state. Instead, it subscribes to a continuous event stream delivered via WebSocket using the STOMP protocol. From the full data stream, only two datapoints are extracted and processed (Table 9).

The script reacts exclusively to changesin datapoint values. If the received colour is identical to the previously stored value, the event is discarded without further processing. This mechanism ensures that only the precise moment of a button press reaches the task queue, rather than a continuous stream of repeated status values.

The embedded HTTP server (localhost:8080) exposes two groups of endpoints, adhering to the principle of interface minimalism (Table 10):

The architecture deliberately constrains the volume of data exchanged between layers. Table 11 summarises this design decision across each interface.

This approach reduces network overhead, simplifies the event-handling logic, and decouples the simulation layer from the physical devices—any KIS.BOX unit can be replaced or reconfigured without modifying the Blender script, provided the datapoint naming convention is preserved.

## 4. Simulation and Discussion

To quantitatively verify the proposed approach, a series of simulation experiments was conducted. Three scheduling algorithms were compared:

- FIFO strategy: A classic greedy rule. Upon the arrival of a task, the algorithm assigns it to the first available robot. If multiple robots are ready, the fastest one is selected. This method does not analyse future system states.
- Rolling window Hungarian Algorithm (HA): a dynamic baseline approach representing a state-of-the-art benchmark for non-predictive, real-time optimal task allocation [3]. While the mathematical foundation of the Hungarian Algorithm is classical, its application within a dynamic, rolling-horizon framework is universally recognized in the AGV scheduling literature as a premier standard for instantaneous dispatching [3]. Because it optimally solves the linear assignment problem in polynomial time, it represents the absolute best-case scenario for any dispatching system that only optimizes for the immediate, current step without a predictive horizon. At each decision step, a cost matrix of size $min\left(n_{r},n_{u}\right)\times min\left(n_{r},n_{u}\right)$ is generated, where $n_{u}$ represents the number of pending tasks. For each robot–task combination, an independent 1-step forward prediction is computed to estimate the task completion time. This estimated time is evaluated using the quadratic function and added to the cumulative $J_{2}$ cost to serve as the corresponding weight in the matrix. A linear assignment optimization is subsequently performed. However, only the optimal assignment for the immediate current task k is executed. The window is then shifted forward, and the process repeats.
- MPC tree: The tree-based algorithm described in Section 2.3, operating with a prediction horizon of $N_{p}=6$.
- FT tree: The fault-tolerant algorithm described in Section 2.4, utilizing a prediction horizon of $N_{p}=6$ and a learning parameter of α=0.7.

It should be noted that both MPC and FT algorithms plan the optimal scheduling sequence and strictly adhere to the execution order of tasks. This means that if a robot assigned to task *k* experiences a delay (e.g., due to a fault), the algorithm halts the system while waiting for its return. Consequently, task k+1 remains blocked, even if another robot assigned to it is ready for dispatch.

The topology of the tested system comprised a single central loading station, $n_{s}=5$ destination stations, and a fleet of $n_{r}=3$ mobile robots. The distances to the respective stations are defined as d={30,25,28,43,33}, while the nominal speeds of the robots are v={1,0.75,0.5}. The state of the predictive algorithms and the robot availability vector x(k) were updated upon the occurrence of two key events: the arrival of a new task and the physical confirmation of a robot’s return to the base station. The task arrival time vector u was generated stochastically to mimic the real-world dynamics of on-demand systems. A set of $N_{u}=30$ tasks was determined based on a base time interval $\Delta T_{u}$. To evaluate the algorithms under various traffic densities, experiments were conducted for three system load scenarios:

1. $\Delta T_{u}=40$: high task frequency;
2. $\Delta T_{u}=50$: moderate task frequency;
3. $\Delta T_{u}=70$: low task frequency.

To introduce realistic deviations from a perfectly periodic rhythm, random noise following a normal distribution $N(0,\sigma^{2})$ with a standard deviation of σ=5 was added to each step. The resulting sequence of arrival times was then sorted chronologically. Destination stations were assigned to each task entirely at random.

The performance of the strategies was evaluated using a quadratic cost function that captures the total time spent by tasks in the system, measured from request arrival to task completion. At each decision step *k*, the MPC algorithms aimed to minimize the following index over the horizon $N_{p}$:

(19) $$\begin{array}{c} J_{2}=\sum_{i=1}^{N_{p}}{\left(y(k+i)-u(k+i)\right)}^{2} \end{array}$$

The application of a nonlinear, quadratic objective function $J_{2}$ aims to balance the production flow. As established in foundational studies on model predictive control for discrete event systems, such as the frameworks developed by van den Boom and De Schutter [14], nonlinear penalty functions provide vital flexibility for complex schedule optimization. While the classic linear criterion $J_{1}=\sum \left(y-u\right)$ is commonly used in related studies utilizing Mixed-Integer Linear Programming (MILP) optimizations [15], it suffers from a critical drawback: it permits the extreme delay of a single task at the expense of minimally accelerating several others. In contrast to these standard linear formulations, the proposed $J_{2}$ function heavily penalizes large individual delays. This design choice explicitly prevents the phenomenon of task “starvation” and ensures an even, stable distribution of assignments among the available robots. The cumulative cost $J_{2}$, computed over all $N_{u}=30$ tasks, was used as the comparison metric. Finally, two disturbance scenarios were considered to test system robustness:

1. Fault-free scenario: No disturbances occurred throughout the entire simulation.
2. Multiple fault scenario:
  - Fault in robot 1 after step k=5: speed reduced to 50% of its nominal value.
  - Obstacle on the path to station 3 after step k=10: distance increased by 20%.
  - Fault in robot 2 after step k=15: speed reduced to 50% of its nominal value.
  - Obstacle on the path to station 1 after step k=15: distance increased by 10%.

**Figure 9:**
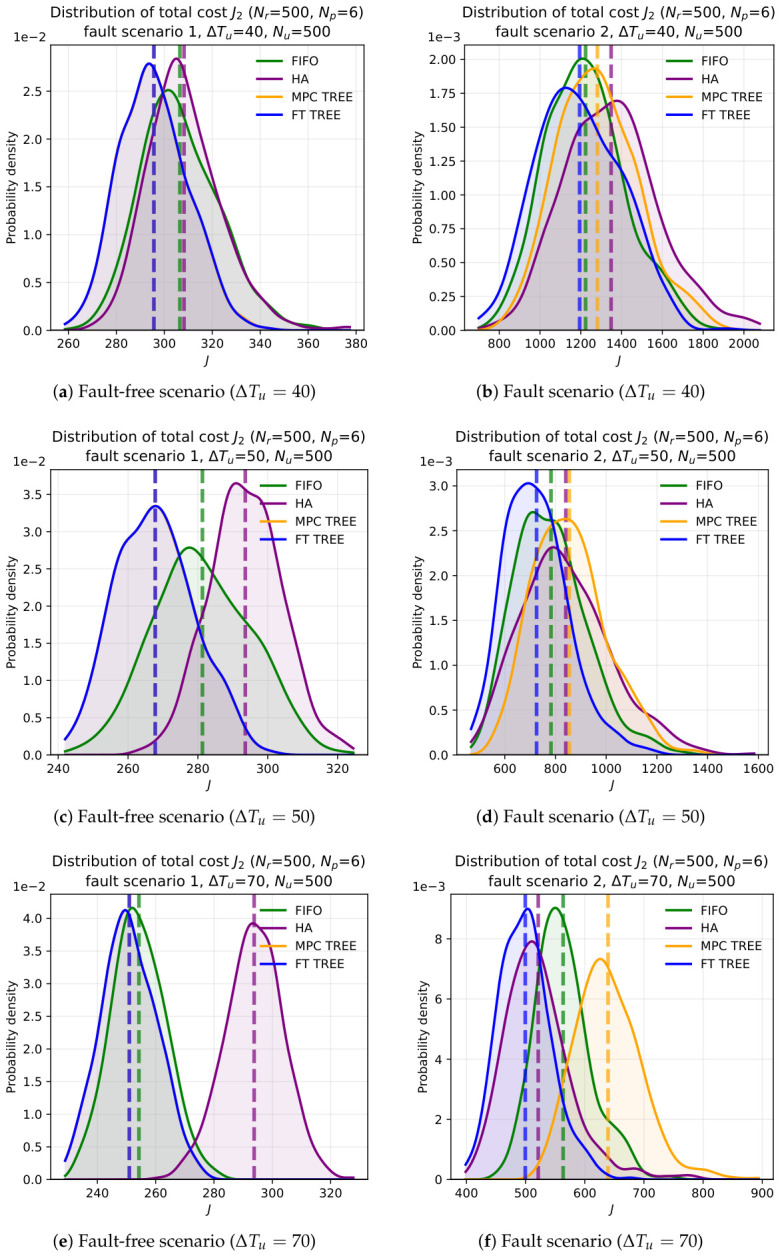
Performance comparison of the FIFO, HA, MPC tree, and FT tree algorithms depending on the task arrival interval ($\Delta T_{u}$) in a fault-free environment and in the presence of disturbances. The vertical dashed lines indicate the average value of the objective function obtained for each method. For (**a**,**c**,**e**), the MPC tree curves are fully overlapped by the FT tree results. The notation 1e−2 denotes $\times 10^{-2}$.

Figure 9 presents a comprehensive summary of the results for $N_{r}=500$ simulation iterations, categorized by system load variants ($\Delta T_{u}$) and the presence of disturbances. The vertical dashed lines in each panel indicate the mean value of the cost function $J_{2}$ for a given algorithm. An analysis of the obtained distributions yields the following conclusions. In the fault-free environment (left column of the plots), the tree-based algorithms (MPC and FT) achieve nearly identical results. On average, they outperform the FIFO strategy. Interestingly, the HA consistently struggles in these scenarios, yielding the highest overall costs. This behaviour stems from the decoupled nature of the Hungarian approximation, which completely neglects the interactions and dependencies between robots. By ignoring the synchronization constraints at the shared loading station ($x_{p}$), HA might create artificial bottlenecks and severe queuing delays. The MPC tree clearly outperforms HA precisely because the employed max-plus algebra explicitly models these interactions, avoiding traffic congestion. Notably, at a low task frequency ($\Delta T_{u}=70$), the performance gap between the tree algorithms and the FIFO approach diminishes. With infrequently arriving tasks, the system possesses a significant time margin. Consequently, the two fastest robots generally manage to complete their missions and return to the base before new requests appear. The slowest unit is engaged only sporadically, serving as a reserve. Under such favourable conditions, long-term planning does not yield substantial optimization gains.

Following the introduction of robot faults and spatial obstacles (right column of the plots), the proposed FT tree algorithm achieves the lowest mean cost $J_{2}$ across all load variants, demonstrating its superiority. The MPC tree struggles due to the previously described blocking phenomenon, as its long time predictions use a wrong, fault-free model. Despite it, the MPC tree outperforms the HA baseline in the highly congested scenario ($\Delta \;T_{u}=40$). Both HA and the MPC tree fall behind even the simple FIFO strategy. This stems from the previously described blocking phenomenon: these algorithms strictly wait for the return of a delayed robot, thereby paralysing the execution of subsequent tasks. FIFO bypasses this problem. The FTC MPC algorithm adapts to the current system state, refining the predictions and consistently producing the best overall outcomes. At a low task frequency ($\Delta \;T_{u}=70$), the advantage of FT tree over the other strategies is most pronounced. The reduced task density provides the algorithm with a sufficient time margin, allowing for flexible schedule reconfiguration. Under heavy task loads ($\Delta T_{u}=40$), the differences between the strategies diminish. Faced with such high demand, every algorithm is forced to dispatch any available robot almost immediately to prevent an uncontrolled growth of the queue.

It should be emphasized that analysing only the distributions and mean cost values does not provide a complete picture of each approach’s performance. Due to the stochastic nature of the generated problem instances, specific circumstances could arise where the simple FIFO strategy performed exceptionally well, while the tree algorithms combined with faults encountered uniquely unfavourable conditions. For this reason, Table 12 introduces a metric of the percentage of iterations won by a given algorithm. This illustrates how often a specific strategy achieved the absolute lowest cost $J_{2}$ within the exact same simulation trial shared across all algorithms. An analysis of these win rates confirms the clear dominance of the FT tree algorithm. In the faulty environment with a lower load ($\Delta T_{u}=70$), it outclasses the competition, winning in 60.8% of the cases. Conversely, the noticeable effectiveness of the FIFO strategy in an extremely overloaded system ($\Delta T_{u}=40$) highlights a critical prerequisite for adaptive methods. To execute effective task reallocation, fault-tolerance mechanisms require the availability of redundant resources within the system. Under full saturation of the robot fleet’s capabilities, the necessary margin of flexibility is absent. The algorithm is deprived of alternative decision paths, which drastically limits its ability to compensate for faults and negates the advantages of advanced planning.

To further dissect the competitive dynamics between the algorithms, Table 13 and Table 14 present the pairwise win-rate matrices for the fault-free and multiple fault scenarios, respectively. This metric evaluates how often a specific strategy achieved a strictly lower cost when directly compared head-to-head against another algorithm. Please note that ties are excluded, so the mutual win rates between any pair of algorithms may sum to less than 100%.

**Figure 10:**
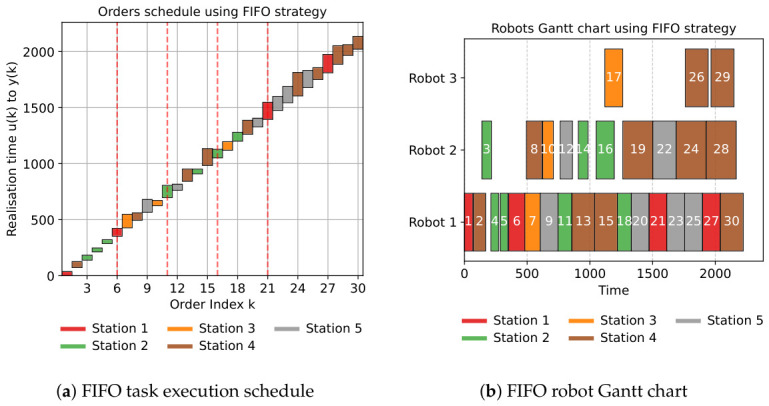

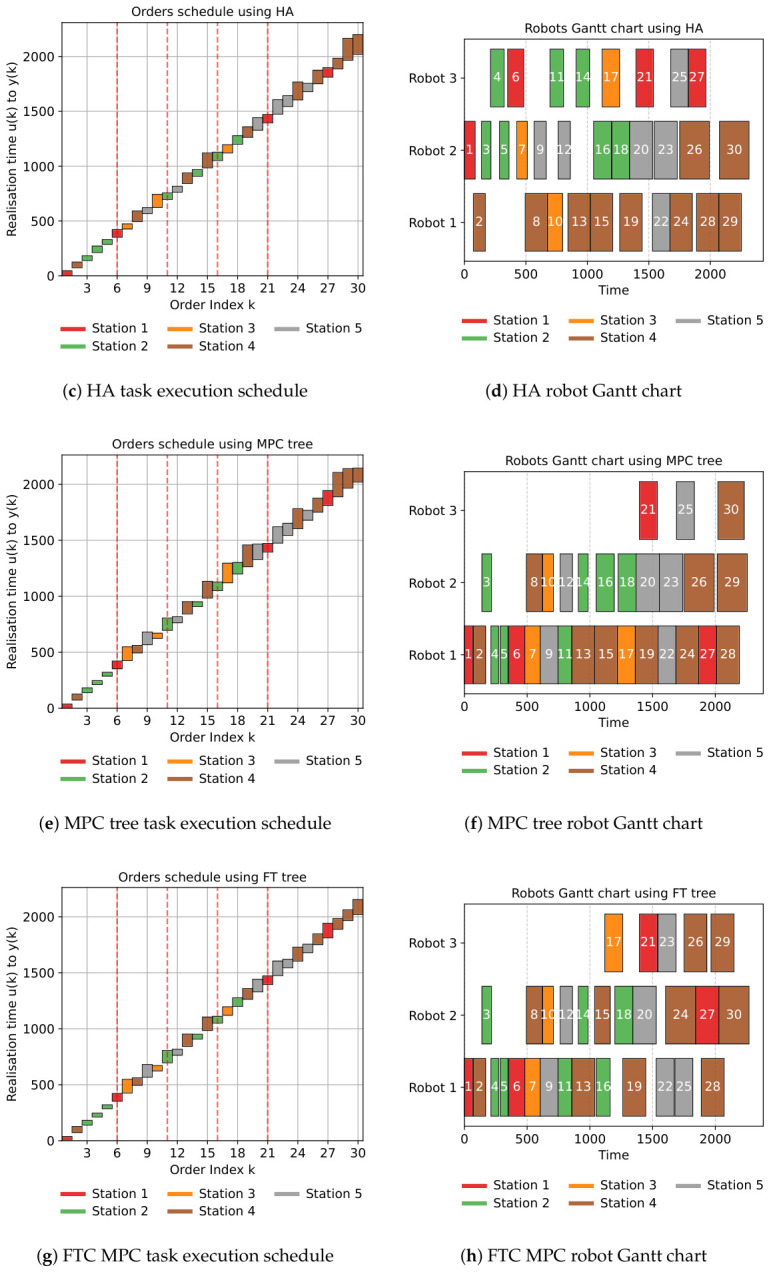
Detailed comparison of temporal characteristics for the disturbed scenario (Fault 2) with $\Delta T_{u}=70$. Red dashed vertical lines indicate the task after which the fault occurs. (Part 1). Detailed comparison of temporal characteristics for the disturbed scenario (Fault 2) with $\Delta T_{u}=70$. Red dashed vertical lines indicate the task after which the fault occurs. (Part 2).

To visualize the differences in task allocation, Figure 10 presents detailed schedules and Gantt charts generated by the evaluated algorithms for a single simulation iteration under the multiple fault scenario ($\Delta T_{u}=70$). The vertical red dashed lines on the plots indicate the exact moments when consecutive disturbances occurred. In the orders schedule plots, the bottom edge of each rectangle represents the moment a task is registered in the system, while the top edge indicates the time of its delivery to the destination station. Furthermore, the Robot Gantt charts illustrate the time intervals during which the robots were engaged in processing specific tasks, spanning from the moment of loading until their return to the base station. The number displayed inside each rectangle corresponds to the index of the executed task *k*.

**Figure 11:**
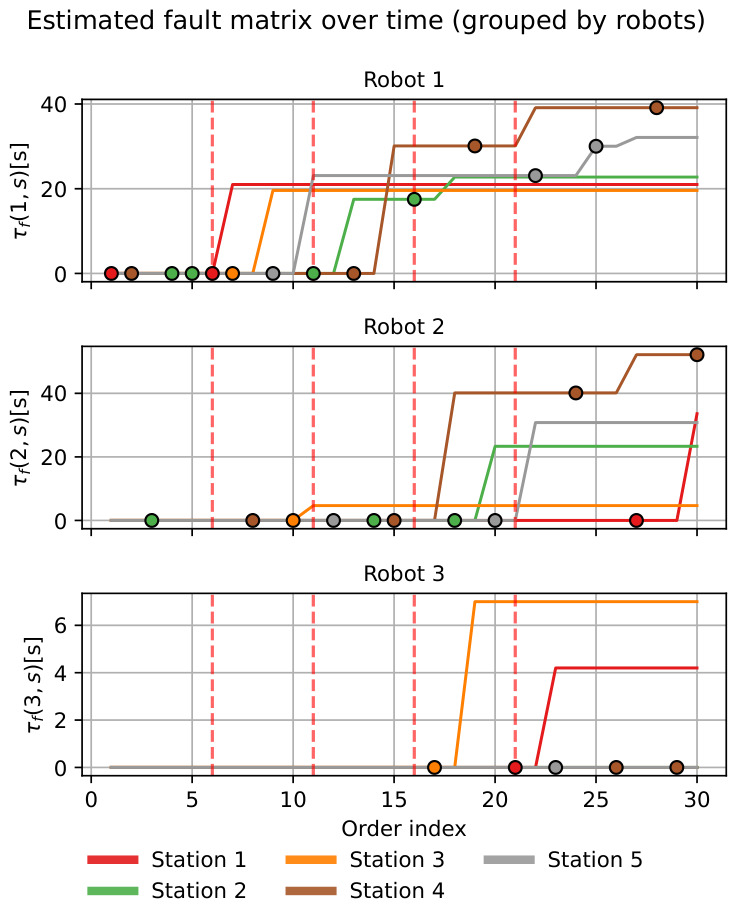
Evolution of the delay estimates produced by the FT tree for $N_{u}=30$ and $\Delta T_{u}=70$. The vertical red dashed lines indicate the fault occurrence times.

**Figure 12:**
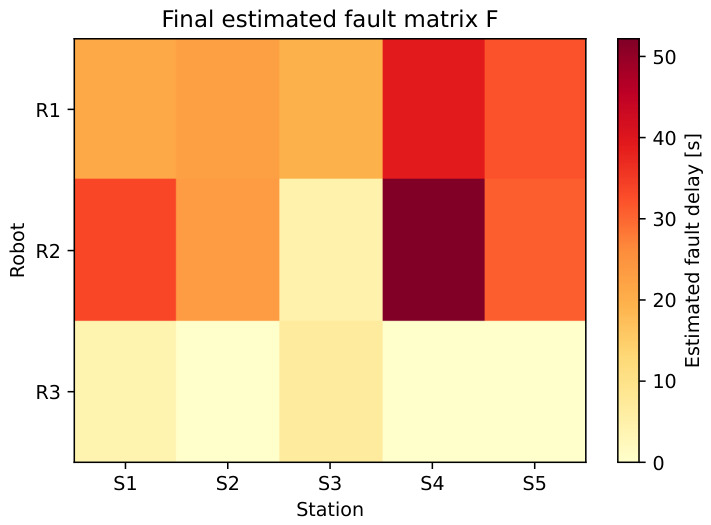
Visualization of the final state of the matrix *F*.

Figure 11 illustrates the evolution of the delay estimates over time (for $N_{u}=30$, $\Delta T_{u}=70$). The circle markers indicate the exact moments of task assignment for a given robot. The update of the estimate is subject to a natural transport delay, as it occurs only after the unit’s physical return to the base station. The trajectories of the curves confirm the convergence of the estimator. Furthermore, Figure 12, which presents a heatmap of the final matrix *F*, demonstrates the diagnostic potential of the algorithm. Analysing the structural correlations within the matrix can help identify the source of the disturbances: an increase in delays across a specific row indicates a hardware fault of a particular robot (e.g., drive degradation), an increase across a column diagnoses an infrastructural anomaly on the route to a destination station (e.g., a permanent obstacle), whereas an anomaly in a single cell signifies a local disturbance in a specific robot–station relation.

## 5. Conclusions

In this paper, an integrated cyber-physical framework for fault-tolerant AGV task scheduling has been presented. By combining a predictive tree search based on switching max-plus linear systems with a physical IoT-based dispatch interface utilizing KIS.BOX devices, the proposed approach successfully addresses both synchronization constraints and non-stationary operational disturbances in modern intralogistics environments.

The quantitative evaluation carried out through extensive simulation experiments demonstrated that the advanced Fault-Tolerant (FT) tree algorithm consistently outperforms traditional scheduling strategies, such as the greedy FIFO rule and the dynamic Rolling-horizon Hungarian Algorithm (HA) baseline. In a fault-free environment, the predictive tree-based methods achieve optimal resource allocation by explicitly modelling robot interactions at the shared loading station, thereby avoiding artificial traffic congestion and bottlenecks. Under multi-fault scenarios involving vehicle degradation and spatial obstacles, the FT tree showcases robustness, achieving the lowest mean cumulative cost ($J_{2}$) and the highest win rate across various traffic densities. Crucially, the integration of a dynamic transport delay estimator ensures rapid adaptation to operational anomalies, while the structural analysis of the final fault matrix *F* offers promising diagnostic capabilities for isolating hardware and infrastructural faults. Ultimately, the results highlight that advanced predictive planning combined with real-time feedback loops is essential for maintaining high operational efficiency and predictability in stochastic manufacturing systems.

Future research will focus on scaling the proposed algorithm to more complex spatial topologies, including multiple centralized loading stations and larger AGV fleets. To mitigate the resulting exponential growth of the decision space, integrating advanced heuristic search methods, such as A* or Monte Carlo Tree Search (MCTS), will be explored for the efficient pruning of suboptimal scheduling branches. Additionally, the predictive model can be extended to incorporate further nonlinear physical constraints, such as fleet energy management and dynamic charging schedules. Finally, the diagnostic potential of the delay estimation matrix could be enhanced by integrating machine learning techniques for the automated classification of specific fault types.

## Figures and Tables

**Table 1:** Comparison of AGV scheduling methodologies. N/A denotes not applicable.

Methodology	Handles Alternative Assignments	Cost Function Flexibility	Fault-Tolerance Mechanism	Real-Time Physical IoT Dispatch
Standard Max-Plus [11,12,14]	No (Static/Cyclic)	N/A	No	No
Max-Plus + MILP [13,15]	Yes	Strictly Linear	No	No
Nominal SMPL Tree [13]	Yes	Nonlinear	No	No
Our Approach (FTC SMPL + IoT)	Yes	Nonlinear	Yes (Adaptive Delay Estimator)	Yes (Event-driven WebSocket/REST)

**Table 2:** Technical specifications of the KIS.BOX device [17].

Parameter	Description
Communication	WLAN (Wi-Fi 2.4 GHz)
Connectors	M12 (8-pin, A-coded)
Power supply	5 V (USB) or 24 V
Inputs/Outputs	2 buttons + 2 GPIO ports (for 24 V)
Protection rating	IP65

**Table 3:** Mapping of LED colours to system values. Colours marked with ★ are actively used as station identifiers in the simulation.

ID	Colour	HEX Code	Active
0	Blue	#0000FF	★
1	Turquoise	#00FFFF	★
2	Black (OFF)	–	
3	Green	#00FF00	
4	Magenta	#FF00FF	★
5	Red	#FF0000	★
6	White	#FFFFFF	
7	Yellow	#FFFF00	★

**Table 4:** Datapoints used in the Blender–KIS.ME integration.

Datapoint	Direction	Type	Description
ledColor	KIS.ME → Blender	Integer (0–7)	Current operational LED colour
ledMode	Blender → KIS.ME	Integer	LED mode: static or flashing
button1	KIS.ME → Blender	Boolean	State of button 1
button2	KIS.ME → Blender	Boolean	State of button 2
deviceStatus	KIS.ME → Blender	String	Device availability status

**Table 5:** Mapping of KIS.ME datapoint events to simulation actions in Blender 3D.

Event (DatapointId)	Value	Action in Blender
button1	true	Advance LED colour to next in sequence
button2	true	Create task, add to queue, lock interface
ledColor	0–7	Update digital twin LED visualisation
deviceStatus	offline	Flag robot as unavailable

**Table 6:** Robot state set.

State	Description
docked	Robot is at its home position, awaiting a task
moving	Robot is travelling to the target location
loading	Loading operation in progress
unloading	Unloading operation in progress
returning	Robot is returning to its home position
fault	Fault state

**Table 7:** Task assignment criteria.

Criterion	Description
Availability	The robot must be idle
Distance	Minimisation of travel distance
Workload	Number of currently assigned tasks
State	The robot must not be in the fault state

**Table 8:** Multi-operator support.

Aspect	Description
Concurrency	Tasks generated simultaneously by multiple operators
Queue	Shared data structure
Priorities	Possibility of task prioritisation

**Table 9:** Datapoints filtered from the KIS.ME event stream.

Datapoint	Description
button1Color	Colour identifier of button 1; a transition from white to any active station colour triggers insertion of a new task into the queue
button2Color	Colour identifier of button 2, encoding the operator-selected target station

**Table 10:** HTTP API endpoints exposed by the Blender script.

Endpoint	Description
GET/status/	Returns a JSON object containing the current state of all robots, KIS.BOX devices, and fault configuration parameters. Intended for external monitoring and decision-tree queries.
GET/{robot}/{destination}	Dispatches the specified robot to the indicated station and schedules its return trip. Accepts optional query parameters speed and station to override defaults at runtime.

**Table 11:** Data minimalism across system interfaces

Interface	Transmitted Data	Omitted Data
KIS.BOX → Blender	Button press colour change	Continuous LED status, device health
Blender → Client	Discrete robot state (5 values)	Full 3D trajectory, interpolated position
Client → Blender	Robot ID + destination	Path planning parameters, timing data

**Table 12:** Detailed statistical summary of simulation results ($N_{r}=500$ iterations for each variant). Won iterations denote the percentage of cases where a given algorithm achieved the lowest value of the cost function $J_{2}$. Bold values indicate the best-performing results.

Fault Scenario	$\Delta T_{u}$	Algorithm	Mean Cost $J_{2}$ (**±*σ***)	Won Iterations
1. (no fault)	40	FIFO	306.50±16.12	2.6%
		HA	308.24±15.34	0.6%
		MPC/FT Tree	295.67±14.00	**96.8%**
	50	FIFO	281.30±13.85	0.8%
		HA	293.58±10.33	0.0%
		MPC/FT Tree	267.79±11.11	**99.2%**
	70	FIFO	254.27±9.21	1.0%
		HA	293.77±9.91	0.0%
		MPC/FT Tree	250.96±9.36	**99.0%**
2. (with faults)	40	FIFO	1223.58±192.55	28.2%
		HA	1348.70±227.11	11.8%
		MPC Tree	1281.48±196.21	14.6%
		FT Tree	1194.25±203.27	**45.4%**
	50	FIFO	782.17±140.99	24.8%
		HA	840.65±175.79	14.8%
		MPC Tree	854.42±143.57	7.6%
		FT Tree	725.33±125.20	**52.8%**
	70	FIFO	563.42±46.88	5.4%
		HA	521.31±56.66	33.8%
		MPC Tree	639.35±55.44	0.0%
		FT Tree	499.40±43.60	**60.8%**

**Table 13:** Pairwise win-rate matrix [%] for scenario 1 (fault-free). Values represent the percentage of iterations where the algorithm in the row achieved a strictly lower cost than the algorithm in the column.

$\Delta T_{u}$	Algorithm (Row vs. Col)	FIFO	HA	MPC/FT Tree
40	FIFO	-	60.8%	2.6%
	HA	39.2%	-	0.6%
	MPC/FT Tree	97.4%	99.4%	-
50	FIFO	-	90.6%	0.8%
	HA	9.4%	-	0.0%
	MPC/FT Tree	99.2%	100.0%	-
70	FIFO	-	100.0%	1.0%
	HA	0.0%	-	0.0%
	MPC/FT Tree	99.0%	100.0%	-

**Table 14:** Pairwise win-rate matrix [%] for fault scenario 2. Values represent the percentage of iterations where the algorithm in the row achieved a strictly lower cost than the algorithm in the column.

$\Delta T_{u}$	Algorithm (Row vs. Col)	FIFO	HA	MPC Tree	FT Tree
40	FIFO	-	71.0%	62.6%	43.0%
	HA	29.0%	-	39.2%	25.2%
	MPC Tree	37.2%	60.8%	-	32.2%
	FT Tree	57.0%	74.8%	67.8%	-
50	FIFO	-	63.2%	69.6%	35.8%
	HA	36.8%	-	54.4%	24.6%
	MPC Tree	30.0%	45.6%	-	19.6%
	FT Tree	64.2%	75.4%	80.4%	-
70	FIFO	-	23.8%	89.6%	12.0%
	HA	76.2%	-	97.4%	36.4%
	MPC Tree	8.2%	2.6%	-	1.2%
	FT Tree	88.0%	63.6%	98.8%	-

## Data Availability

The original contributions presented in this study are included in the article. Further inquiries can be directed to the corresponding author.
